# Effect of Molybdenum on the Impact Toughness of Heat-Affected Zone in High-Strength Low-Alloy Steel

**DOI:** 10.3390/ma14061430

**Published:** 2021-03-15

**Authors:** Xiaoyan Wu, Pengcheng Xiao, Shujing Wu, Chunliang Yan, Xuegang Ma, Zengxun Liu, Wei Chen, Liguang Zhu, Qingjun Zhang

**Affiliations:** 1 Hebei Provincial High-Quality Steel Continuous Casting Engineering Technology Research Center, College of Metallurgy and Energy, North China University of Science and Technology, Tangshan 063210, China; wuxy@stu.ncst.edu.cn (X.W.); xiaopc@ncst.edu.cn (P.X.); liuzengxun@ncst.edu.cn (Z.L.); hblgdxzzb@163.com (W.C.); 2 College of Science, North China University of Science and Technology, Tangshan 063210, China; wushujing@xidian.edu.cn; 3 Analysis and Testing Center, North China University of Science and Technology, Tangshan 063210, China; yanchunliang66@163.com (C.Y.); 13653255957@126.com (X.M.); 4 School of Materials Science and Engineering, Hebei University of Science and Technology, Shijiazhuang 050018, China

**Keywords:** molybdenum, microstructural evolution, heat-affected zone, impact toughness, precipitates, high-strength low-alloy steel

## Abstract

The microstructure, precipitates, and austenite grain in high-strength low-alloy steel were characterized by optical microscope, transmission electron microscope, and laser scanning confocal microscopy to investigate the effect of Mo on the toughness of steel. The microstructure was refined and the toughness was enhanced after the addition of 0.07% Mo in steel. The addition of Mo can suppress the Widmanstätten ferrite (WF) formation and promote the transformation of acicular ferrite (AF), leading to the fine transformed products in the heat-affected zone (HAZ). The chemical composition of precipitates changed from Nb(C, N) to (Nb, Mo)(C, N) because of the addition of Mo. The calculated lattice misfit between Nb(C, N) and ferrite was approximately 11.39%, while it was reduced to 5.40% for (Nb, Mo)(C, N), which significantly affected the size and number density of precipitates. A detailed analysis of the precipitates focusing on the chemical composition, size, and number density has been undertaken to understand the contribution of Mo on the improvement of steel toughness.

## 1. Introduction

Owing to the superior comprehensive properties, including excellent low-temperature impact toughness, high tensile strength, and good weldability, high-strength low-alloy (HSLA) steels have been widely applied in various fields, such as ship construction, offshore engineering, navy vessels, and even nuclear fission power plant components [1,2,3,4,5,6,7,8,9]. In recent years, in order to reduce the construction cost and enhance the welding efficiency, high heat input welding rises in response to the proper time and conditions. However, the heat-affected zone (HAZ) of the steel plate must be subjected to a higher temperature thermal cycle and longer residence at high temperature during high heat input welding [10]. Consequently, high heat input invariably leads to significant coarsening of austenite grains and formation of brittle microstructures, such as ferrite side plate (FSP), Widmanstätten ferrite (WF), and coarsening grain boundary ferrite (GBF), which significantly reduces the toughness of HAZ [11,12]. In particular, the coarse-grained heat-affected zone (CGHAZ) adjacent to the welding fusion line, incurred by rapid heating and varied cooling rate with higher peak temperature, always shows the worst impact toughness. Therefore, how to improve the toughness of HAZ has become an important research topic as the strength, plate thickness, and welding heat input continue to increase.

The refinement of grains by the pinning effect of fine precipitates has been acknowledged as an effective way to improve the toughness and strength of steel [13,14,15]. The migration and growth of prior austenite grain after the high-temperature welding thermal cycle in the HAZ can be inhibited by a large number of fine carbides and nitrides [16,17]. As a well-known microalloying addition, Nb, V, Ti, and Mo could refine the grain size and provide fine carbonitride precipitates, which facilitate superior mechanical properties [18,19,20,21]. Gómez et al. reported that the addition of Nb in steel was beneficial for retarding the static recrystallization of austenite and achieving fine ultimate microstructures [22]. Medina et al. pointed out that VN precipitates can lead to a significant decrease in the grain size in a high nitrogen content vanadium micro-alloyed steel [23]. The stable TiN precipitates have also been demonstrated to be the most effective micro-alloyed precipitation particles for inhibiting the growth of austenite [24]. As a matter of fact, extensive efforts have been dedicated to studying the contribution of the precipitates containing Nb, Ti, and V, but little research focuses on the effect of Mo on the precipitates and toughness of the steel. Moreover, it still remains unclear the precise role of Mo in the precipitation behavior of pinning particles. The experimental evidence on the effects of Mo on precipitates and toughness of steel should be explored further.

In this study, four kinds of steel with different Mo content were designed to systematically investigate the effect of Mo on the toughness of steel. The microstructural evolution of cast ingots, hot-rolled steel sheets, and CGHAZ was analyzed by optical microscope (OM). The macroscopic and microscopic characteristics of fracture were examined to further analyze the toughness of steel. The changes in chemical composition, size, and number density of precipitates and their contribution to the refinement of austenite size and improvement of toughness were elaborated in detail.

## 2. Materials and Methods

### 2.1. Material Preparation

**Figure 1:**
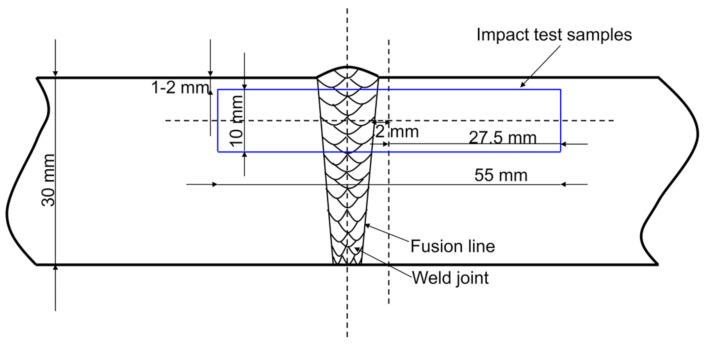
Schematic diagram of the machining locations of Charpy V-notch impact samples.

The four kinds of studied steels containing no Mo (N1), 0.04% Mo (N2), 0.06% Mo (N3), and 0.07% Mo (N4) were smelted in a 100 kg vacuum induction furnace and cast into ingots. The chemical compositions of four kinds of steel were determined using optical emission spectroscopy (OES-5500, Shimadzu, Kyoto, Japan) and given in Table 1. Subsequently, the ingots were heated to 1200 °C in a heating furnace and held for two hours to fully austenitize. The ingots were hot rolled to a thickness of 30 mm by six passes. The temperature of the ingots taken out of the heating furnace was 1180 °C, the starting rolling temperature was 1160 °C, and the finishing rolling temperature was 830 °C. The parameters about the degree of deformation during hot rolling are shown in Table 2. The 30-mm-thick steel sheet was machined into a V-shaped groove with an included angle of 45°. The welding test was carried out with the American Lincoln three-wire submerged arc welding machine (Lincoln Electric, Cleveland, OH, USA). A single-pass submerged-arc welding process was employed with a heat input of approximately 100 kJ/cm. Afterward, the steel sheets were machined into standard “V” notch Charpy impact test samples with dimensions of 10 × 10 × 55 mm^3^. The samples were subjected to impact tests at the distance of 2 mm from the fusion line at −20 °C by a drop-weight impact tester (JB-W300A series). Figure 1 presents the schematic diagram of the machining locations of Charpy V-notch impact samples. A total of five samples were prepared for testing the impact toughness in each of the studied steel. Generally, three samples were selected for the test, and the other two were reserved as alternative samples. When the deviation of test value was large, the alternative samples were used for testing. Finally, three impact test values with small deviations were selected, and the average value was taken as the impact test result.

### 2.2. Material Characterization

The samples were chemically etched in a 3% nitric acid solution (3 mL HNO_3_ + 97 mL C_2_H_5_OH) for 25 s after mechanical polishing. The observation of microstructure in steel was performed by Leica OM. FEI scanning electron microscope (SEM, Scios, FEI, Hillsboro, OR, America) and Leica stereomicroscope were conducted to analyze the morphological characteristics of macroscopic and microscopic fracture. The four kinds of steel sheets were machined into cylindrical samples with a diameter of 7 mm and a height of 3 mm, which were austenitized at 1100 °C for 300 s and cooled to room temperature at a cooling rate of 7 °C/s by laser scanning confocal microscopy (LSCM, VL2000DX-SVF17SP, Lasertec, Kanagawa, Japan) to in situ record and observe the grain growth of austenite. A JEOL JEM-2800 transmission electron microscope (TEM, JEOL, Toyoshima, Japan) equipped with energy-dispersive X-ray spectroscopy (EDS) detection system was employed to determine the sizes and chemical compositions of precipitates. The samples for TEM observation were prepared by the carbon extraction replica technique. To prepare carbon extraction replicas, the polished samples were etched with 2% nitric acid solution (2 mL HNO_3_ + 98 mL C_2_H_5_OH), followed by evaporation of carbon evaporated onto the etched surface. Finally, the surface was scored to ≈ 3 mm squares and the sample etched first with 10% nitric acid solution (10 mL HNO_3_ + 90 mL C_2_H_5_OH) and then soaked with alcohol. Subsequently, the extracted replicas were rinsed with distilled water, placed on the copper grid and dried. A substantial number of measurements were conducted to determine the average size of the precipitates. To investigate the characteristics of precipitates in steel, scanning transmission electron microscope (STEM) bright-field (BF)/dark-field (DF) images, EDS mappings of precipitates in steel were acquired by using TEM equipped with an EDS detection system operated at a voltage of 200 kV.

## 3. Results and Discussion

### 3.1. Microstructural Evolution

**Figure 2:**
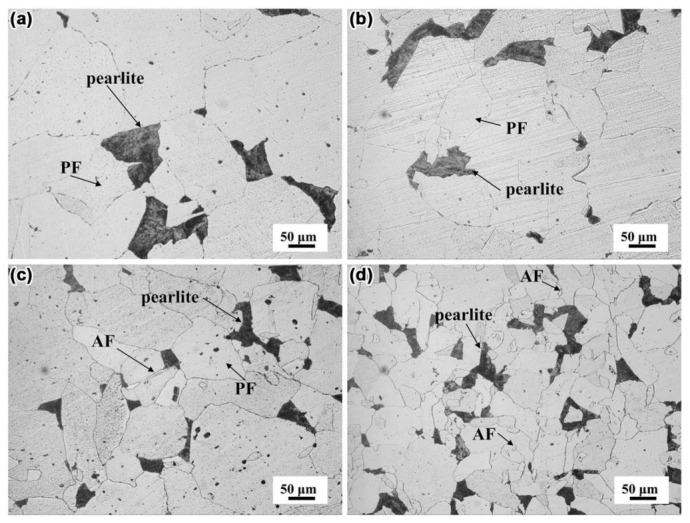
The microstructure of cast ingots, (**a**) N1, (**b**) N2, (**c**) N3, and (**d**) N4 steel.

**Figure 3:**
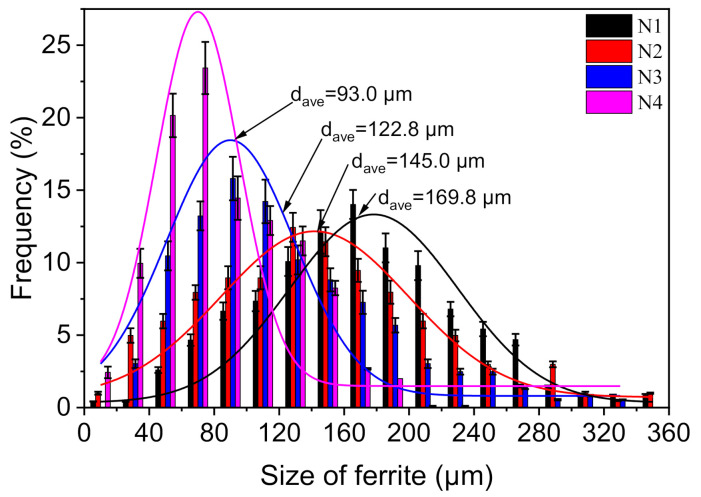
Size distribution of ferrite grains in the four kinds of cast ingot.

To investigate the effect of Mo content on the microstructure, the microstructure of the cast ingots was observed by OM. Figure 2a–d shows the microstructure of the cast ingots of N1, N2, N3, and N4 steel, respectively. The microstructures in cast ingots were composed of ferrite and pearlite. In the N1 steel without Mo and N2 steel with 0.04% Mo, the microstructures of cast ingots were composed of polygonal ferrite (PF) and pearlite (Figure 2a,b). When the content of Mo was 0.06%, the size of ferrite grain was significantly refined, and a small amount of fine acicular ferrite (AF) was observed (Figure 2c). As a contrast, with 0.07% Mo, the size of ferrite grain was uniform and further refined (Figure 2d). The size distribution of ferrite grains in the four kinds of cast ingot is shown in Figure 3. The overall size distribution gradually shifted to the left as Mo was added to the steel, and thus, the ferrite grains exhibited a decreasing tendency in size. Moreover, the size distributions of N1, N2, N3, and N4 steel follow the log-normal form, with average sizes of 169.8, 145.0, 122.8, and 93.0 μm, respectively. It means that with the increase of Mo content in the range of 0–0.07%, the size of ferrite grain has a tendency to decrease.

**Figure 4:**
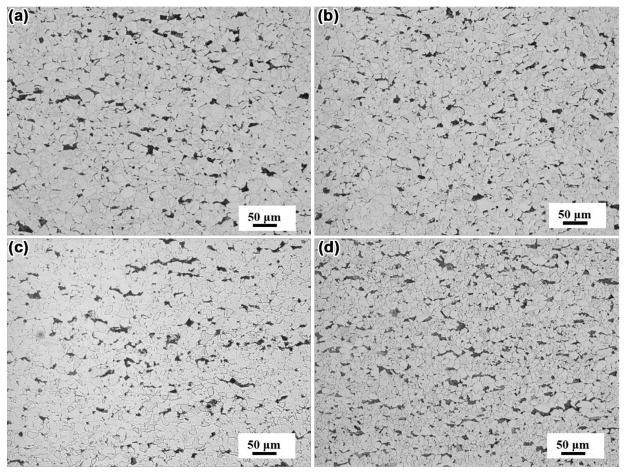
The microstructure of hot rolled steel sheets, (**a**) N1, (**b**) N2, (**c**) N3, and (**d**) N4 steel.

**Figure 5:**
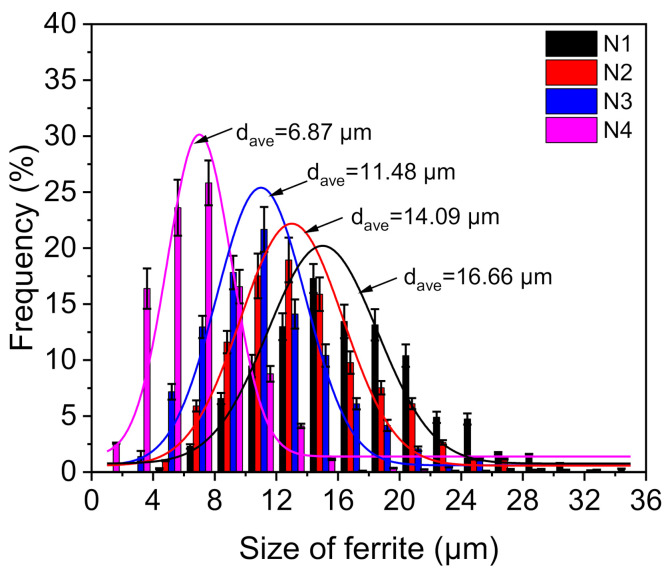
Size distribution of ferrite grains in the four kinds of rolled steel sheet.

Figure 4 shows the microstructure characteristics of hot rolled steel sheets. The microstructure of hot rolled steel sheets of N1, N2, N3, and N4 steel was shown in Figure 4a–d, respectively. The size distribution of ferrite grains in the four kinds of rolled steel sheet is shown in Figure 5. The overall size distribution gradually shifted to the left as Mo was added to the steel, and thus, the ferrite grains exhibited a decreasing tendency in size. Moreover, the size distributions of N1, N2, N3, and N4 steel follow the log-normal form, with average sizes of 16.66, 14.09, 11.48, and 6.87 μm, respectively. These results indicated that an amount of addition of Mo led to significant grain refinement in the cast ingots and rolled steel sheets, which may be beneficial for the improvement of the steel properties.

**Figure 6:**
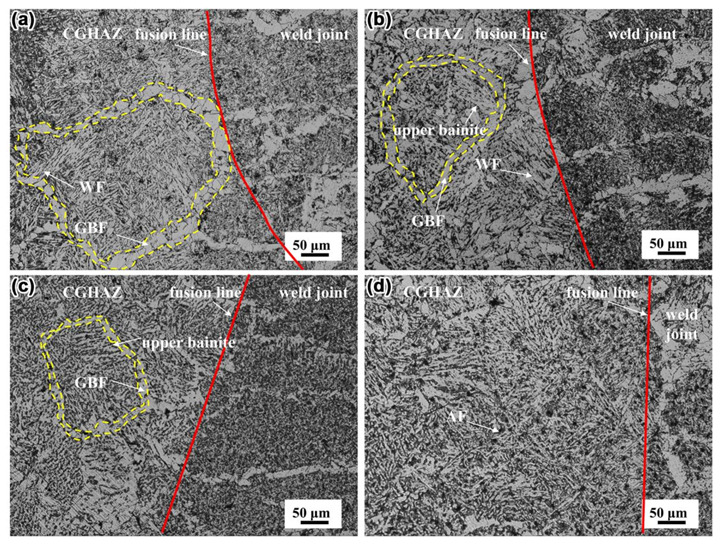
The microstructure of coarse-grained heat-affected zone (CGHAZ) in steels, (**a**) N1 steel, (**b**) N2 steel, (**c**) N3 steel, and (**d**) N4 steel.

To study the effect of Mo on the impact toughness of HAZ in steel, the microstructure characteristics of CGHAZ after single submerged arc welding were studied by OM. Figure 6a–d shows the microstructure of CGHAZ in N1, N2, N3, and N4 steel, respectively. As shown in Figure 6a, the coarse GBF and WF were observed in CGHAZ of N1 steel. The microstructure in N2 steel containing 0.04% Mo consisted of GBF, WF and upper bainite (Figure 6b), and the size of GBF and austenite were finer than those in N1 steel. As shown in Figure 6c, although a small amount of upper bainite was observed, the size of GBF and austenite were further refined in the N3 steel containing 0.06% Mo. The austenite in N1, N2, and N3 steel was outlined by the yellow dotted line, as shown in Figure 6a–c. The width of GBF in CGHAZ of N1, N2, and N3 steel was approximately 18.72, 10.71, and 8.24 μm, respectively, which indicated that it decreased with the increase of Mo content. It is likely that the size of austenite in CGHAZ of N1, N2, and N3 steel was approximately 275, 221, and 165 μm, respectively, which also decreased with the increase of Mo content. The microstructure in CGHAZ was mainly comprised of AF when 0.07% Mo was added in steel (Figure 6d). The addition of Mo reduced the formation of GBF and WF that were detrimental to toughness and enhanced the transformation of fine AF that contributed to the improvement of toughness [25]. These results suggested that the addition of Mo can strongly influence the microstructure in CGHAZ, thereby affecting the impact toughness of HAZ. In other words, further improvement of the impact toughness of HAZ in steel may be achieved by the addition of Mo.

### 3.2. Impact Toughness of Steels

**Figure 7:**
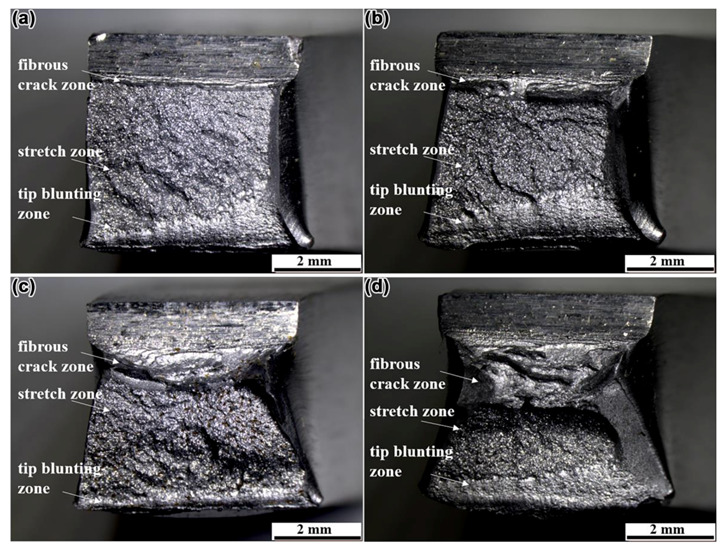
The macroscopic morphology of fracture after impact test at the distance of 2 mm from the fusion line at −20 °C, (**a**) N1 steel, (**b**) N2 steel, (**c**) N3 steel, and (**d**) N4 steel.

The macroscopic morphology of fracture was examined by stereomicroscope. Figure 7a-d shows the macroscopic morphology of fracture in N1, N2, N3, and N4 steel, respectively. It indicates that the macroscopic morphology of the fracture surface of the samples with different Mo content is quite different when they are subjected to the same impact test conditions. Figure 7a shows the fracture surface of the sample without Mo addition. The surface of the fracture was flattening and had obvious metallic luster. The stretch zone covered a large proportion of the total area of the fracture. Figure 7b shows the fracture surface of the sample with 0.04% Mo addition. Compared with the stretch zone in Figure 7a, the area of the stretch zone in Figure 7b was reduced. Figure 7c shows the fracture surface of the sample with 0.06% Mo addition. The area of the fibrous crack zone increased significantly, while the area of the stretch zone decreased further. As shown in Figure 7d, the area of the fibrous crack zone and tip blunting zone increased significantly, and the fracture surface was dark and had no metallic luster. As the content of Mo in the steel increased, the area of the fibrous crack zone and tip blunting zone continued to increase, and the fracture surface fluctuated more and more. It has been pointed out in the literature that the longer the fibrous crack zone extends, the better the toughness of the material [26]. With the increase of Mo content, the proportion of fibrous crack zone and tip blunting zone in the fracture increased, and the proportion of stretch zone was correspondingly reduced, which suggested that the toughness may be enhanced by the appropriate addition of Mo in steel.

**Figure 8:**
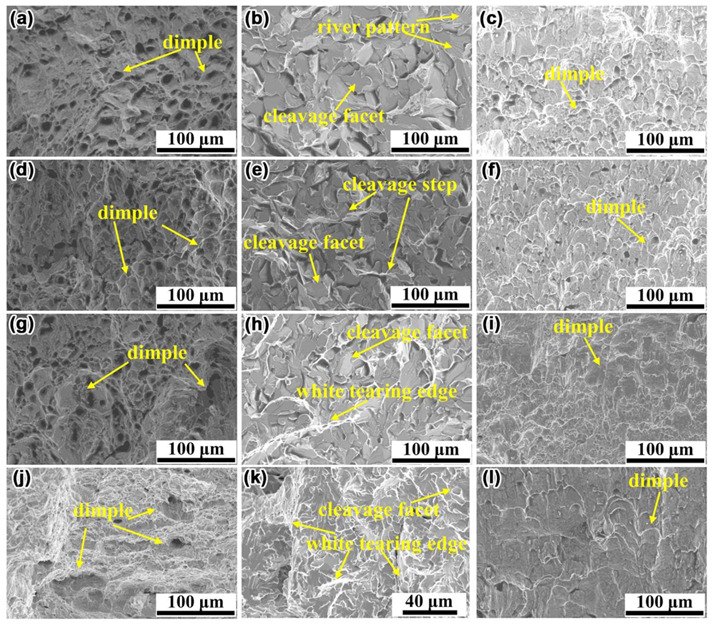
The micromorphology of the fibrous crack zone (**a**,**d**,**g**,**j**), the stretch zone (**b**,**e**,**h**,**k**), and the tip blunting zone (**c**,**f**,**i**,**l**) in N1, N2, N3, and N4 steel, respectively.

The micromorphology of fracture was further characterized by SEM. Figure 8 shows the micromorphological characteristics of the fracture in N1, N2, N3, and N4 steel. The micromorphology of the fibrous crack zone in the four kinds of steel was shown in Figure 8a,d,g,j, respectively. Round or oval dimples of different sizes were densely distributed in the fibrous crack zone. Figure 8b,e,h,k shows the micromorphology of the stretch zone in N1, N2, N3, and N4 steel, respectively. There were a few river patterns on the cleavage facets (Figure 8b). With the addition of Mo, the height of cleavage steps increased (Figure 8e). As shown in Figure 8h, white tearing edges were observed in the stretch zone of the fracture, and the cleavage surface was uneven, indicating that the crack propagation path was tortuous. In Figure 8k, the cleavage facets were smaller, more white tearing edges were observed, and the cleavage surface was further uneven, which indicated that the steel possessed the ability of a certain resistance to crack propagation. It can be inferred from Figure 8b,e,h,k that with the increase of Mo content, a lot of deformation and crack direction transformation occurred during crack propagation, which showed a certain excellent toughness. The morphology of the tip blunting zone in N1, N2, N3, and N4 steel was shown in Figure 8c,f,i,l, respectively. It can be seen from Figure 8c,f,i,l that the tip blunting zones in the fracture were densely covered with dimples. With the increase of Mo content, the size of the dimple gradually tended to increase. The analysis of fracture micromorphology further showed that the micromorphology of fracture changed greatly with the increase of Mo content, which suggested that the morphology characteristics of fracture may be closely related to the toughness of the steel.

The standard “V” notch Charpy impact test samples were subjected to impact tests at the distance of 2 mm from the fusion line at −20 °C by a drop-weight impact tester. The impact test scheme and the testing results obtained from the HAZ in the four kinds of steel are displayed in Table 3. The average value of impact energy of the HAZ in N1, N2, N3, and N4 steel sheets was 70, 108, 155, and 170 J, respectively. In the range of 0−0.07%, the impact energy of HAZ increased with the increase of Mo content, which indicated that the addition of an appropriate amount of Mo was beneficial to improve the impact toughness of HAZ in steel. The test results of impact toughness in Table 3 were in good agreement with the analysis results of fracture morphology in Figure 7 and Figure 8. When Mo was not added into the steel, the stretch zone occupied a large area and the impact toughness was low (Figure 7). As the content of Mo increased, the area occupied by the fibrous zone and the tip blunting zone gradually increased, which was accompanied by a gradual increase in impact toughness. Therefore, it was believed that the macroscopic and microscopic morphological characteristics of the fracture can be used to estimate the impact toughness of the steel.

### 3.3. Effect of Mo Addition on the Precipitation Behavior

#### 3.3.1. Austenite Size in Steels

**Figure 9:**
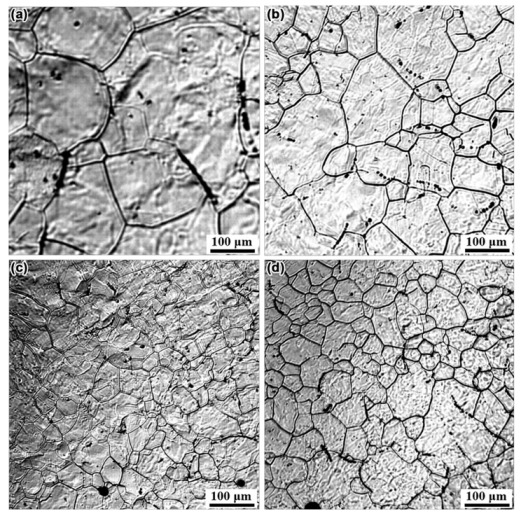
In situ observation of austenite grain size in N1 (**a**), N2 (**b**), N3 (**c**), and N4 steel (**d**).

**Figure 10:**
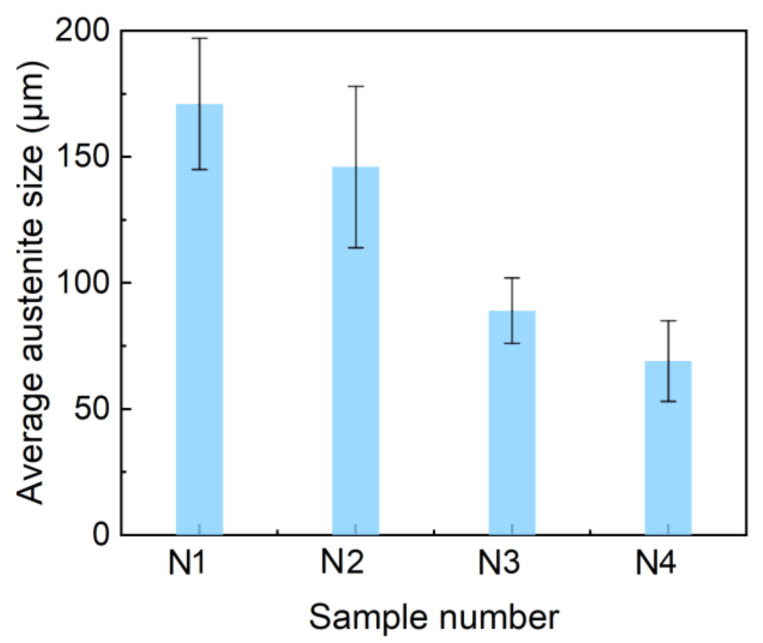
The average size of austenite grain in N1, N2, N3, and N4 steel.

The changes of austenite size in the four kinds of steel were in situ observed and recorded by LSCM to evaluate the role of Mo in the refinement of microstructure. The samples prepared for LSCM observation were austenitized at 1100 °C for 300 s. Typical results reflecting austenite grain size characteristics in N1, N2, N3, and N4 steel are shown in Figure 9a–d, respectively. The equivalent diameter of 400 austenite grains in N1, N2, N3, and N4 steel was measured and statistically analyzed, respectively. According to the statistical results, the average size of austenite grain in N1, N2, N3, and N4 steel was obtained. The average size of austenite in N1 steel without Mo addition, N2 steel with 0.04% Mo, N3 steel with 0.06% Mo, and N4 steel with 0.07% Mo was 171, 146, 89, and 69 μm, respectively. The changes in the average size of austenite in the four kinds of steel are presented in Figure 10, which indicates a pronounced tendency of decreasing in the average size of austenite with the increase of Mo content. This result further supported that the addition of Mo can refine the grain, which may be associated with the pinning effect of the precipitates located in the grain boundaries [27,28].

#### 3.3.2. Precipitates in Steels

**Figure 11:**
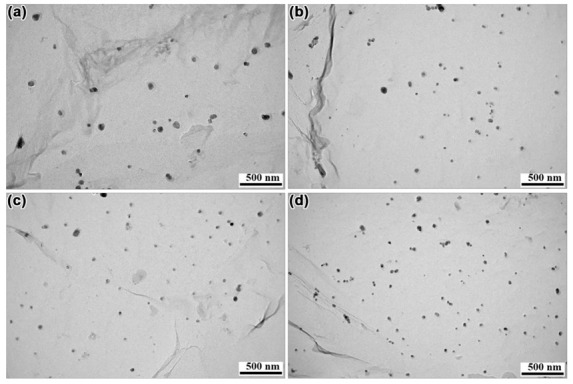
The precipitates in the four kinds of steel, (**a**) N1, (**b**) N2, (**c**) N3, and (**d**) N4 steel.

The chemical composition, size distribution, and number density of precipitates in steel were investigated by TEM. The samples obtained from the base material after rolling were austenitized at 1100 °C for 300 s and then used for TEM observation by the carbon extraction replica technique. The distribution of precipitates in the four kinds of steel is displayed in Figure 11. The size and number density of precipitates differed from steel to steel and also as a function of the difference in Mo content. Fifty images showing the distribution of the precipitates in each steel were obtained by TEM, which allowed us to count the number of precipitates per area. The number density of the precipitates in N1, N2, N3, and N4 steel was 5 × 10^6^/mm^2^, 1.2 × 10^7^/mm^2^, 1.8 × 10^7^/mm^2^, and 3.5 × 10^7^/mm^2^, respectively. It was obviously observed that the number density of precipitates showed a continuous increasing trend with the increase of Mo content.

**Figure 12:**
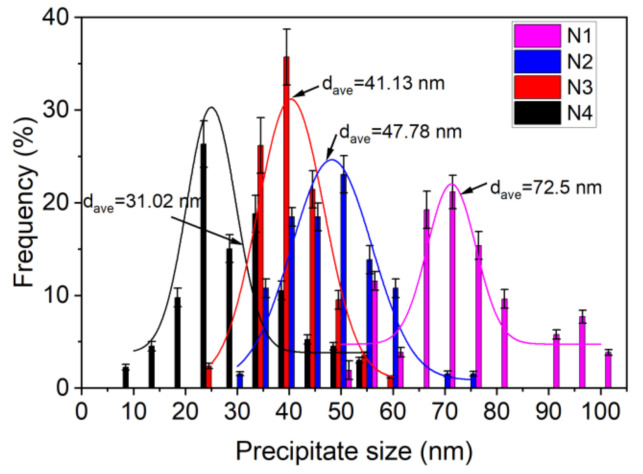
Size distribution of the precipitates in the four kinds of steel.

The size of 400 precipitates in N1, N2, N3, and N4 steel were measured by Image J software, respectively. The size distribution of precipitates in the four kinds of steel is shown in Figure 12. The overall size distribution gradually shifted to the left as Mo was added to the steel, and thus, the precipitates exhibited a decreasing tendency in size. Moreover, the size distributions of N1, N2, N3, and N4 steel follow the log-normal form, with average sizes of 72.5, 47.78, 41.13, and 31.02 nm, respectively. The size corresponding to the peak value in N1, N2, N3, and N4 steel was 67.5–72.5 nm with a proportion of 21.15%, 47.5–52.5 nm with a proportion of 23.08%, 37.5–42.5 nm with a proportion of 35.71%, and 22.5–27.5 nm with a proportion of 26.32%, respectively. These results revealed that the addition of Mo resulted in a significant increase in the number density and a reduction in the size of precipitates.

**Figure 13:**
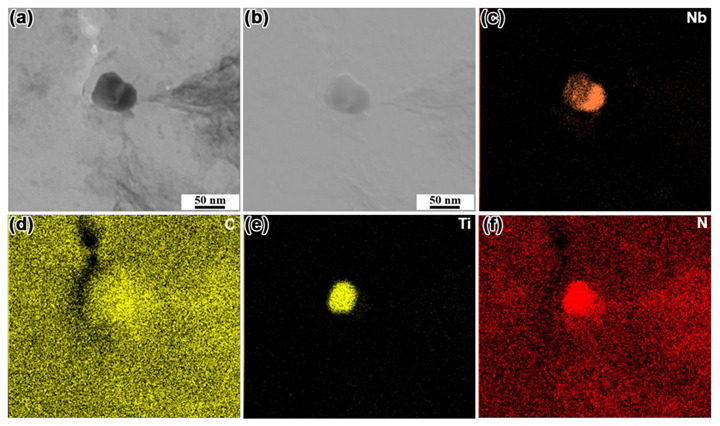
(**a**) STEM bright-field (BF) image of the typical precipitate, (**b**) STEM dark-field (DF) image of the typical precipitate, (**c**–**f**) energy-dispersive X-ray spectroscopy (EDS) mapping of the precipitate in the N1 steel without Mo addition.

**Figure 14:**
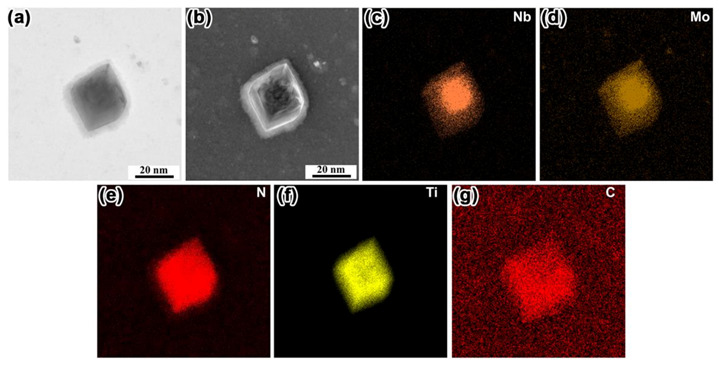
(**a**) STEM BF image of the typical precipitate, (**b**) STEM DF image of the typical precipitate, (**c**–**g**) EDS mapping of the precipitate in the N4 steel with 0.07% Mo addition.

The chemical composition of precipitates in steel was determined by TEM equipped with EDS detection to study the role of Mo in the changes of number density and size of precipitates. Figure 13 shows the morphology and elemental distribution of the typical precipitates in N1 steel without Mo. Figure 13a,b shows the STEM BF and STEM DF image of the precipitate, respectively. The corresponding EDS mappings indicated that the precipitate was mainly composed of Nb, C, Ti, and N elements, i.e., Ti (C, N) + Nb (C, N). Furthermore, Ti (C, N) and Nb(C, N) adhered to each other and precipitated. Figure 14 shows the morphology and elemental distribution of the typical precipitates in N4 steel with 0.07% Mo. The morphology characteristic of the precipitate is presented in Figure 14a,b. The precipitates consisted of Nb, Mo, C, Ti, and N elements. Further observation showed that the distribution of Mo was almost the same as that of Nb. Mo, as the nearest neighbor element in the same period of Nb, has a close atomic number to Nb: 42 and 41, respectively. Thus, it can be inferred that with the addition of Mo to the steel, the complex precipitate phase (Nb, Mo)(C, N) was formed by the partial replacement of Nb by Mo in the Nb(C, N). The precipitates with square, spherical or irregular morphology were observed by TEM in N1, N2, N3, and N4 steel. Based on the results of EDS mapping, it can be inferred that the morphology of precipitates was related to the distribution of their composition. The precipitates turned out to be a square shape, when Nb(C, N) or (Nb, Mo)(C, N) was present in the core, and Ti (C, N) was distributed at the Nb(C, N) or (Nb, Mo)(C, N) periphery (Figure 14). However, the morphology of precipitates was similar to spherical or irregular shape when Ti (C, N) and Nb(C, N) or (Nb, Mo)(C, N) adhered to each other and precipitated (Figure 13).

**Figure 15:**
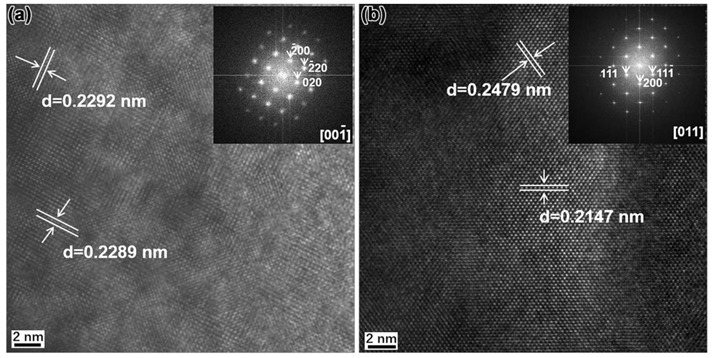
(**a**) HRTEM image of Nb(C, N) in N1 steel, (**b**) HRTEM image of (Nb, Mo)(C, N) in N4 steel. The corresponding Fast Fourier Transform (FFT) images are shown in the inset.

Figure 15a,b show the high-resolution TEM (HRTEM) image of Nb(C, N) in N1 steel and (Nb, Mo)(C, N) in N4 steel, respectively. According to the HRTEM image and Fast Fourier Transform (FFT) of precipitates, it can be concluded that the lattice parameters of Nb(C, N) and (Nb, Mo)(C, N) are 0.458 nm and 0.429 nm, respectively. It is believed that because of the addition of Mo, the precipitates changed from Nb(C, N) to (Nb, Mo)(C, N), which caused the decrease in the lattice parameter of precipitates. Within the allowable range of measurement and calculation errors, the calculated results were consistent with those reported in previous literature [29].

It has been reported that the carbonitride was believed to hold a Baker–Nutting orientation relationship with the ferrite matrix [30]. The lattice misfit between ferrite and carbonitride can be calculated by the following equation [29]:

(1) $$\delta =\frac{a_{carbonitride}-\sqrt{2}a_{\alpha }}{a_{carbonitride}}$$

where δ is the lattice misfit between ferrite and carbonitride, $a_{carbonitride}$ is the lattice parameter of carbonitride, and $a_{\alpha }$ is the lattice parameter of ferrite. In view of $a_{\alpha }$= 0.287 nm [31], the lattice misfit (δ) between ferrite and Nb(C, N) was approximately 11.39%, while the δ between ferrite and (Nb, Mo)(C, N) was approximately 5.40% calculated by Equation (1). This means that the partial replacement of Nb by Mo resulted in the reduction of lattice misfit, which in turn caused the decrease in the interfacial energy and enhanced the nucleation rate of the precipitates at the early stage of nucleation. Thus, the increase in the number density of precipitates was attributed to the decrease of lattice parameter caused by the addition of Mo. Moreover, in the range of 0–0.07%, a further increase in the number density of the precipitates can be achieved by the further increase in the Mo content.

As to the coarsening rate of precipitates, it can be described by the following equation [32]:

(2) $$m={\left(\frac{8\sigma V_{p}^{2}Dc_{0}}{9V_{B}c_{p}RT}\right)}^{1/3}$$

where m is the coarsening rate of the precipitates, σ is the interface energy between ferrite and precipitates, $V_{p}$ is the molar volume of the precipitates, D is the diffusion coefficient of the controlling elements, $c_{0}$ is the solute atomic concentration of the controlling elements, $V_{B}$ is the molar volume of the solute element, and $c_{p}$ is the solute atomic equilibrium concentration of the controlling elements. According to Equation (2), the m value decreased as the σ value decreased. It can be inferred that the decrease of interface energy caused by the partial replacement of Nb by Mo can refine the size of precipitates. Additionally, Pan et al. had pointed out that the size of (Nb, Mo)(C, N) was smaller than that of Nb(C, N) [32]. Therefore, due to the addition of Mo, the average size of precipitates was reduced significantly, and the number density of precipitates was increased substantially.

It has been demonstrated that a relatively small change in the number density and size distribution of the precipitates can strongly influence the size of ferrite and prior austenite grain [27]. The effect of precipitates on the size of ferrite and austenite grain can be explained by Zener’s theory that the driving force for grain growth due to the curvature of the grain boundary can be counteracted by a pinning effect exerted by particles. The pinning effect can be described by the following equation [33]:

(3) $$D_{c}=\frac{\pi d}{6f}(\frac{3}{2}-\frac{2}{Z})$$

where $D_{c}$ is the size of grain, d is the average diameter of the precipitates, f is the volume fraction of precipitates, and Z is a constant. The value of f can be calculated by the following equation [34]:

(4) $$f=\frac{\pi Nd^{2}}{6S_{0}}$$

where N is the quantity of particles per area, and $S_{0}$ is the specific area for estimation. According to Equation (4), it can be calculated that the volume fraction of precipitates in N1, N2, N3, and N4 steel was 1.375%, 1.433%, 1.593%, and 1.761%, respectively. With the increase of Mo content, the volume fraction of precipitates tended to increase. When the volume fraction f and diameter d of precipitates was substituted into Equation (3), the size of grain ($D_{c}$) in N1, N2, N3, and N4 steel can be calculated as follows: 2759.40 (1.5–2/Z), 1745.17 (1.5–2/Z), 1351.21 (1.5–2/Z), and 922.11 (1.5–2/Z), respectively. It can be inferred that $D_{c}$ tended to decrease when Z is a constant. Thus, Equation (4) indicated that the finer size and higher volume fraction of precipitates had a more positive pinning effect on the grain, which was consistent with the change trend obtained from the experimental results. It is believed that the increase in the number density and decrease in the size of precipitates caused by the addition of Mo can refine the size of grains. The grain refinement is known to be conducive to the improvement of toughness. Therefore, the improvement of the toughness, through the refinement of grains, can be achieved by the addition of Mo.

## 4. Conclusions

In summary, the effect of Mo on the microstructure, precipitates, and impact toughness in HAZ were investigated in this study. For the range of 0–0.07%, the addition of Mo gave a reduction in the size of grains. Meanwhile, the addition of Mo retarded the transformation of WF in CGHAZ and promoted AF formation. With the enhancement of the impact toughness in HAZ, the area of fibrous crack zone increased gradually, and the area of the stretch zone was correspondingly reduced. Due to the addition of Mo, the precipitates were changed from Nb(C, N) to (Nb, Mo)(C, N). Furthermore, the addition of Mo not only increased the number density of precipitates but also diminished the size, which in turn promoted the refinement of the grain size and the enhancement of the steel toughness. The decrease of lattice misfit resulted from the partial replacement of Nb by Mo was responsible for the increase in the number density of precipitates. A refinement in the size of precipitates was attributed to the reduction of the interface energy between the precipitates and ferrite.

## Figures and Tables

**Table 1:** Chemical composition of the steels (wt %).

Samples	C	Si	Mn	P	S	Mo	Al	Ti	Nb
N1	0.06	0.23	1.44	0.016	0.004	0	0.032	0.017	0.032
N2	0.06	0.21	1.43	0.015	0.006	0.04	0.030	0.013	0.037
N3	0.06	0.23	1.41	0.015	0.006	0.06	0.037	0.017	0.035
N4	0.06	0.24	1.60	0.015	0.008	0.07	0.030	0.018	0.034

**Table 2:** The parameters about the degree of deformation during hot rolling.

Thickness of steel/mm	100	85	70	55	45	35	30
Reduction of per pass/mm	0	15	15	15	10	10	5
Reducing rate/%	0	15	17.6	21.4	18.2	22.2	14.3

**Table 3:** The impact test scheme and impact energy of the heat-affected zone (HAZ) in steel.

Samples	V-notch Location/mm	Test Temperature/°C	Impact Energy/J	Average Value of Impact Energy/J
N1	Fusion line + 2	−20	70	70
			76	
			64	
N2	Fusion line + 2	−20	103	108
			115	
			107	
N3	Fusion line + 2	−20	140	155
			160	
			165	
N4	Fusion line + 2	−20	178	170
			159	
			173	

## Data Availability

All data generated or analyzed during this study are included in this article.

## References

[1] Vervynckt S., Verbeken K., Lopez B., Jonas J.J. (2012). Modern HSLA steels and role of non-recrystallisation temperature. Int. Mater. Rev..

[2] Jun H.J., Kang K.B., Park C.G. (2003). Effects of cooling rate and isothermal holding on the precipitation behavior during continuous casting of Nb–Ti bearing HSLA steels. Scripta Mater..

[3] Han G., Xie Z.J., Xiong L., Shang C.J., Misra R.D.K. (2017). Evolution of nano-size precipitation and mechanical properties in a high strength-ductility low alloy steel through intercritical treatment. Mat. Sci. Eng. A.

[4] Charleux M., Poole W.J., Militzer M., Deschamps A. (2001). Precipitation behavior and its effect on strengthening of an HSLA-Nb/Ti steel. Metall. Mater. Trans. A.

[5] Shao Y., Liu C., Yan Z., Li H., Liu Y. (2018). Formation mechanism and control methods of acicular ferrite in HSLA steels: A review. J. Mater. Sci. Technol..

[6] Lee K.H., Kim M.C., Lee B.S., Wee D.M. (2010). Master curve characterization of the fracture toughness behavior in SA508 Gr.4N low alloy steels. J. Nucl. Mater..

[7] Bu F.Z., Wang X.M., Yang S.W., Shang C.J., Misra R.D.K. (2015). Contribution of interphase precipitation on yield strength in thermomechanically simulated Ti–Nb and Ti–Nb–Mo microalloyed steels. Mat. Sci. Eng. A.

[8] Franceschi M., Pezzato L., Gennari C., Fabrizi A., Polyakova M., Konstantinov D., Brunelli K., Dabalà M. (2020). Effect of intercritical annealing and austempering on the microstructure and mechanical properties of a high silicon manganese steel. Metals.

[9] Franceschi M., Pezzato L., Settimi A.G., Gennari C., Pigato M., Polyakova M., Konstantinov D., Brunelli K., Dabalà M. (2021). Effect of different austempering heat treatments on corrosion properties of high silicon steel. Materials.

[10] Zou X., Sun J., Matsuura H., Wang C. (2020). Unravelling microstructure evolution and grain boundary misorientation in coarse-grained heat-affected zone of EH420 shipbuilding steel subject to varied welding heat inputs. Metall. Mater. Trans. A.

[11] Pu J., Yu S.F., Li Y.Y. (2017). Effects of Zr-Ti on the microstructure and properties of flux aided backing submerged arc weld metals. J. Alloy Compd..

[12] Kojima A., Yoshii K., Hada T., Saeki O., Ichikawa K., Yoshida Y., Shimura Y., Azuma K. (2004). Development of high HAZ toughness steel plates for box columns with high heat input welding. Nippon. Steel Tech. Rep..

[13] Wan X., Zhou B., Nune K.C., Li Y., Wu K., Li G. (2017). In-situ microscopy study of grain refinement in the simulated heat-affected zone of high-strength low-alloy steel by TiN particle. Sci. Technol. Weld. Joi..

[14] Chen X.W., Qiao G.Y., Han X.L., Wang X., Xiao F.R., Liao B. (2014). Effects of Mo, Cr and Nb on microstructure and mechanical properties of heat affected zone for Nb-bearing X80 pipeline steels. Mater. Design.

[15] Chen C.Y., Chen C.C., Yang J.R. (2014). Microstructure characterization of nanometer carbides heterogeneous precipitation in Ti–Nb and Ti–Nb–Mo steel. Mater. Charact..

[16] Yan B., Liu Y., Wang Z., Liu C., Si Y., Li H., Yu J. (2017). The Effect of Precipitate Evolution on Austenite Grain Growth in RAFM Steel. Materials.

[17] Hu J., Du L.X., Zang M., Yin S.J., Wang Y.G., Qi X.Y., Gao X.H., Misra R.D.K. (2016). On the determining role of acicular ferrite in V-N microalloyed steel in increasing strength-toughness combination. Mater. Charact..

[18] Sun J., Lu S. (2020). Influence of inter-dendritic segregation on the precipitation behaviour and mechanical properties in a vanadium-containing Fe–Cr–Ni–Mo weld metal. Scripta Mater..

[19] Hu H., Xu G., Wang L., Xue Z., Zhang Y., Liu G. (2015). The effects of Nb and Mo addition on transformation and properties in low carbon bainitic steels. Mater. Design.

[20] Gong P., Liu X.G., Rijkenberg A., Rainforth W.M. (2018). The effect of molybdenum on interphase precipitation and microstructures in microalloyed steels containing titanium and vanadium. Acta Mater..

[21] Jang J.H., Heo Y.U., Lee C.H., Bhadeshia H.K.D.H., Suh D.W. (2013). Interphase precipitation in Ti–Nb and Ti–Nb–Mo bearing steel. Mater. Sci. Tech-lond.

[22] Gómez M., Medina S.F., Quispe A., Valles P. (2002). Static recrystallization and induced precipitation in a low Nb microalloyed steel. ISIJ Int..

[23] Medina S.F., Gómez M., Rancel L. (2008). Grain refinement by intragranular nucleation of ferrite in a high nitrogen content vanadium microalloyed steel. Scripta Mater..

[24] Prikryl M., Kroupa A., Weatherly G.C., Subramanian S.V. (1996). Precipitation behavior in a medium carbon, Ti-V-N microalloyed steel. Metall. Mater. Trans. A.

[25] Costin W.L., Lavigne O., Kotousov A. (2016). A study on the relationship between microstructure and mechanical properties of acicular ferrite and upper bainite. Mat. Sci. Eng. A.

[26] Cao R., Li G., Fang X.Y., Song J., Chen J.H. (2013). Investigation on the effects of microstructure on the impact and fracture toughness of a C–Mn steel with various microstructures. Mat. Sci. Eng. A.

[27] Zhang Y., Li X., Liu Y., Liu C., Dong J., Yu L., Li H. (2020). Study of the kinetics of austenite grain growth by dynamic Ti-rich and Nb-rich carbonitride dissolution in HSLA steel: In-situ observation and modeling. Mater. Charact..

[28] Mandal G.K., Das S.S., Kumar T., Kamaraj A., Mondal K., Srivastava V.C. (2018). Role of precipitates in recrystallization mechanisms of Nb-Mo microalloyed steel. J. Mater. Eng. Perform..

[29] Jang J.H., Lee C.H., Heo Y.U., Suh D.W. (2012). Stability of (Ti,M)C (M=Nb, V, Mo and W) carbide in steels using first-principles calculations. Acta Mater..

[30] Lee W.B., Hong S.G., Park C.G., Park S.H. (2002). Carbide precipitation and high-temperature strength of hot-rolled high-strength, low-alloy steels containing Nb and Mo. Metall. Mater. Trans. A.

[31] Li X., Wang Z., Deng X., Li Y., Lou H., Wang G. (2016). Precipitation behavior and kinetics in Nb-V-bearing low-carbon steel. Mater. Lett..

[32] Pan H., Ding H., Cai M. (2018). Microstructural evolution and precipitation behavior of the warm-rolled medium Mn steels containing Nb or Nb-Mo during intercritical annealing. Mat. Sci. Eng. A.

[33] Ming L., Wang Q., Wang H., Zhang C., Wei Z., Guo A. (2014). A remarkable role of niobium precipitation in refining microstructure and improving toughness of A QT-treated 20CrMo47NbV steel with ultrahigh strength. Mat. Sci. Eng. A.

[34] Wang Q., Zhang C., Li R., Gao J., Wang M., Zhang F. (2013). Characterization of the microstructures and mechanical properties of 25CrMo48V martensitic steel tempered at different times. Mat. Sci. Eng. A.

